# The IlluminOss® photodynamic bone stabilization system for pathological osteolyses and fractures of the humerus: indications, advantages and limits in a series of 12 patients at 24 months of minimum follow‐up

**DOI:** 10.1186/s12891-020-03927-6

**Published:** 2021-01-11

**Authors:** Carmine Zoccali, Dario Attala, Mattia Pugliese, Alessandra Scotto di Uccio, Jacopo Baldi

**Affiliations:** 1 Oncological Orthopaedics Department, IRCCS - Regina Elena National Cancer Institute, Via Elio Chianesi 53, 00144 Rome, Italy; 2 Orthopedics and Traumatology School, Department of Surgery, Sapienza University of Rome, Piazzale Aldo Moro 3/5, 00100 Rome, Italy; 3 School of General Surgery, Hepatobiliary Surgery and Organ Transplant Unit, Department of Surgery, Sapienza University of Rome, Piazzale Aldo Moro 3/5, 00100 Rome, Italy

**Keywords:** Humeral fracture, Impending fracture, Intramedullary stabilization, Osteosynthesis, Osteoplasty, Bone metastasis

## Abstract

**Background:**

Locked titanium nails are considered the reference treatment for metastatic bone lesions of the humerus in patients with aggressive histotypes, high risk of fracture or when estimated survival is lower than 6 months.Nevertheless, they are responsible for CT and MRI artifacts which interfere with postoperative radiotherapy and follow-up.The IlluminOss® is an intramedullary stabilization system which is introduced inside the humeral canal in a deflated state, and is then distended with a monomer which hardens after exposure to blue light,stabilizing the segment; it does not cause artifacts, allowing easier and more effective radiotherapy and follow-up.

The aim of this study is to report our experience, indications, possible advantages and limitations of this stabilization system at 24 months of minimum follow-up in a series of 12 patients affected by pathological fractures or impending fractures of the humerus.

**Methods:**

This is a retrospective case-series that included all patients who underwent surgery with the IlluminOss® Photodynamic Bone Stabilization System for pathological osteolyses and fractures of the humerus. Intraoperative and postoperative complications were valued.

**Results:**

12 patients and 13 procedures were included in the study. All surgeries were performed without intraoperative complications. No early postoperative complications were noted. The wounds healed in all cases and stitches were removed at two weeks from surgery, so the patients were able to perform chemotherapy after three weeks. All patients except one had a painless active range of motion which reached 90°.The VAS score was 7 preoperatively and 2.6 at one month from surgery. Pain relief was also associated to radiotherapy and chemotherapy.

Unfortunately, two nail ruptures were reported at 4 and 12 months of follow-up.

No artifacts were noted in the postoperative CT scans so the radiotherapy plans were easily performed without the need of dose compensation.

**Conclusions:**

The IlluminOss® intramedullary stabilization system can provide primary stability in humeral fractures and impending fractures;the surgical technique is easy and minimally invasive.Moreover,it does not present artifacts at postoperative imaging,probably giving a better chance to perform prompt radiotherapy and chemotherapy.However, randomized clinical studies are necessary to verify its potential strength and if precocious adjuvant radio- and chemotherapy are associated to a reduction of the local progression rate.

## Background

The humerus is the most common site for metastatic bone lesions, after the spine and the femur [1, 2].

Even if the lesions are usually lytic, only 8 to 10% of them evolve into a fracture or an impending fracture and pose indications for a preventive treatment in case of impending fractures or a stabilization in case of fractures that have already occurred [3, 4].

Nevertheless, pathological humeral fractures are associated with a low quality of life and conservative treatment shows poor results because the tumor destroys the bone callus and the fracture tends not to heal; because of these factors, surgery has to be carefully valued and may be proposed more often [5].

Indication for surgical treatment depends moreover on survival, which is influenced by several factors such as the aggressiveness of the primary tumor, the patient’s general conditions, presence of further metastases and their localization, and the availability of effective systemic therapies; risk of fracture and possible surgical techniques also have to be taken into consideration [6, 7].

While resection and prosthetic reconstruction is suggested when estimated survival is higher than 24 months, intramedullary (IM) stabilization is considered the mainstay treatment in patients with aggressive histotypes, high risk of fracture or when estimated survival is lower than 6 months.

Locked titanium nails are considered the reference treatment [8, 9]. Carbon nails are a viable alternative, allowing an easier follow-up because of the reduced metal-related artifacts and the minimal dose perturbation and refractory dispersion effects. Moreover, they allow a better adjunctive radiotherapy because target volume centering is improved and, theoretically, a lower irradiation dose is needed to hit the therapeutic threshold [1, 10].

The recent introduction of a new, minimally invasive IM system can provide low-impact surgery plus an easier and safer post-operative radiotherapy [2, 11].

The IlluminOss® photodynamic Bone Stabilization System (IS-PBSS) is a minimally invasive technique consisting in a balloon catheter which is inserted in the medullary canal uninflated and is then filled with a monomer to completely pervade the canal, adhering to the internal surface of the cortical bone; a blue light is then applied from an external source through an optical fiber, inducing polymerization and making the system harden. It is suitable both for diaphyseal and metaphyseal osteolyses and fractures by virtue of its customized size and its adhesion to the surrounding bone [12]. The aim of this study is to report our experience, indications, possible advantages and limitations of the use of the IS-PBSS at 24 months of minimum follow-up in a series of 12 patients affected by pathological osteolyses or impending fractures of the humerus.

## Methods

All patients who underwent surgical stabilization for pathological fractures or impending fractures of the humerus using the IS-PBSS (IlluminOss Medical, Inc., East Providence, RI, USA) from September 2014 to December 2017 in an oncological research hospital were included in the present series.

Criteria for IS-PBSS IM stabilization were: fractures or impending fractures of the diaphysis of the humerus in intermediate or radiosensitive histologies, in patients affected by multiple metastases with an estimated survival shorter than 24 months in case of solid tumors and independently from survival in case of multiple myeloma, and with a sufficient bone stock. In case of osteolysis, Mirel’s criteria were applied to evaluate the risk of fracture, with a score greater than 8 suggesting prophylactic fixation [1, 13].

Exclusion criteria were fractures or impending fractures on radioresistant histologies, estimated survival longer than two years for solid tumors, a longer than 5 cm discontinuity of the bone segment; in those cases, resection and prosthetic reconstruction was considered more indicated.

All patients were strictly evaluated by a multidisciplinary team including an expert oncological orthopedic surgeon, a radiologist, a medical oncologist, a radiotherapist and an anesthesiologist to identify the best therapeutic indications.

Survival was estimated considering the histology, the number of bone and visceral metastases, the availability of systemic therapies and the patient’s performance status.

The American Society of Anesthesiologist (ASA) score was used to value the anesthetic risk.

When the estimated survival was less than three months or the ASA score was ≥ 4 the lesion was treated conservatively [14].

Before surgeries, an X-ray and an MRI were performed in all cases to value the extension of the disease and the possible presence of other metastases in the same segment.

Cephazolin (20–30 mg/kg) was administered prior to surgery and every eight hours for the subsequent 24 h, as part of antibiotic prophylaxis.

### Surgical procedure

**Fig. 1:**
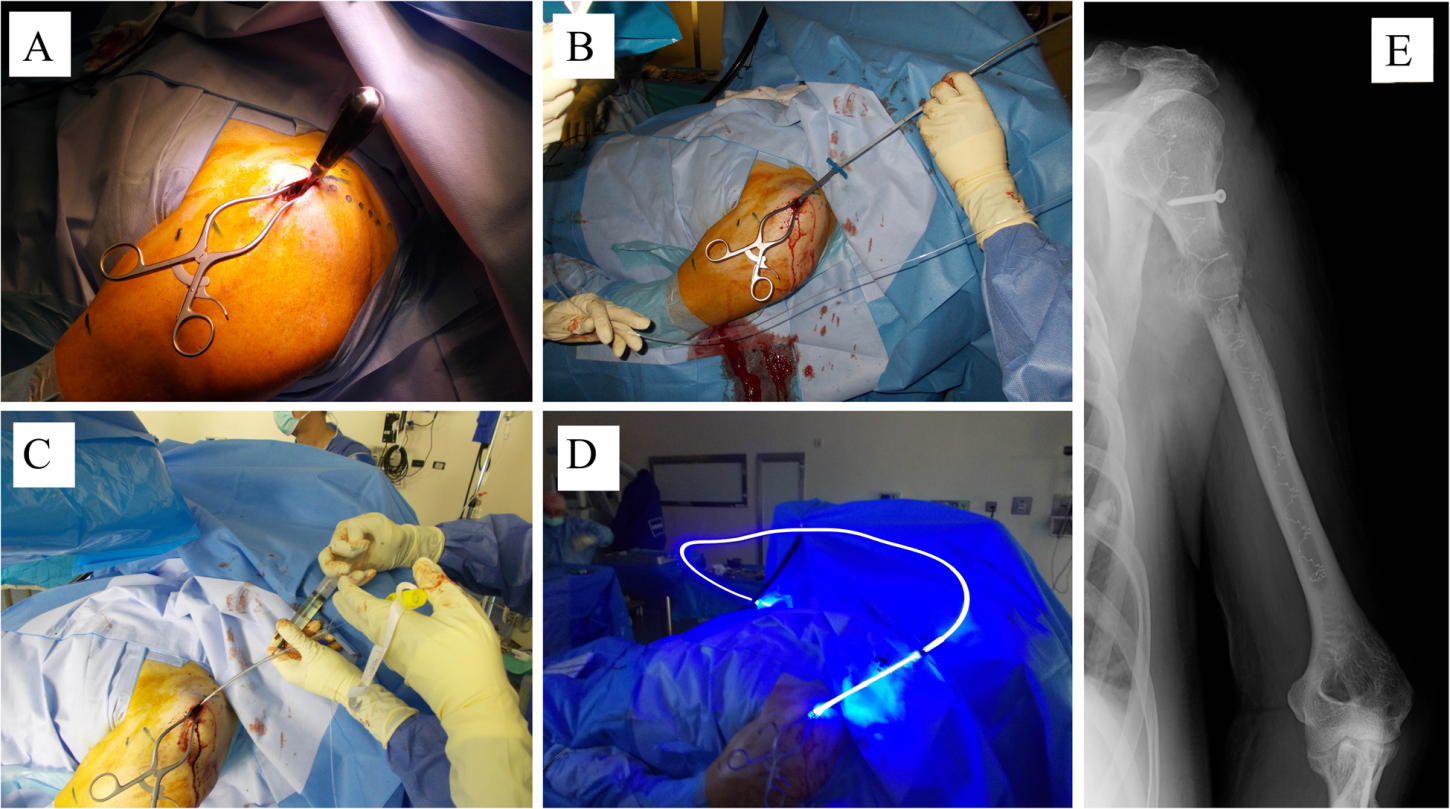
**a**: the patient is placed in beach-chair position, a minimally invasive incision is perform just laterally or anteriorly to the acromion and a sharp instrument is used to gain the intramedullary canal; **b**: the fluid nail is then inserted in the medullary canal in a deflated state; **c**: the balloon is filled with the monomer until a complete expansion is obtained (verified by an increase of the resistance in the syringe or by an X-Ray intensifier) ; **d**: the polymerization process is activated by the blue light; **e**: the postoperative X-ray showing the nail correctly expanded as demonstrated by the helicoidal markers; a supplementary screw was used to increase the stability of the proximal segment

The technique is similar to traditional nailing, despite some intra-operative differences due to the implant’s radiolucency. Markers are present to facilitate the identification of the balloon (Fig. 1e).

The patient is placed on the operative table in a “Beach Chair” position or, alternatively, on the back with the upper body elevated at a 30° angle. A radiolucent table or a removable shoulder support are necessary, as the entire humerus (from elbow to shoulder) must be visible under fluoroscopy. The fractured arm must be supported on a side rest.

After sterile field preparation, a 2 cm incision is made on the anterolateral side of the acromion process, splitting the deltoid muscle longitudinally (Fig. 1a). The greater tuberosity is palpated and the rotator cuff incised in line with the medullary canal to allow IS-PBSS insertion in a straight axis, without reaming the canal (Fig. 1b). The entry point can be variable since it is a flexible delivery system with a small diameter; indeed, a more lateral approach may also be possible, sparing the rotator cuff.

The IS-PBSS is based on the use of a thin-walled polyethylene terephthalate (PET) Dacron balloon with a diameter ranging from 7 to 17 mm with 2 mm increments, or 22 mm; after insertion, it is infused with a liquid monomer (Fig. 1c); as the balloon is filled with the monomer, it conforms to the specific shape of the patient’s bone. Once in position, a source of visible 436 NM light is activated inside the balloon, thus initiating the polymerization and the hardening of the monomer (Fig. 1d).

During polymerization, an exothermic reaction increases local temperature, which reaches a peak of 62 °C for 24 seconds, then 49 °C for 108 seconds, 38 °C for 100 seconds and finally sets at body temperature.

Once hardened, the implant provides longitudinal and rotational stability throughout its entire length as it has modelled itself to the shape of the patient’s IM canal – the result is stabilization of the fracture similar to that of a traditional IM nail. Likewise, if the obtained stability is deemed insufficient, the nail can be drilled and screws can be inserted in any point in order to give the system additional rigidity, especially if the fracture line lies close to the extremities of the nail (Fig. 1e).

The upper limb was maintained in an arm sling for seven days, allowing Codman pendulum exercises. After seven-ten days from surgery, progressive passive motions were performed below 90 degrees of forward flexion and lateral abduction. At two weeks, progressive active exercises were permitted.

The patients were followed-up at 1, 3, 6, 9, 12 and 18 months from surgery and then every 12 months with an X-ray.

**Fig. 2:**
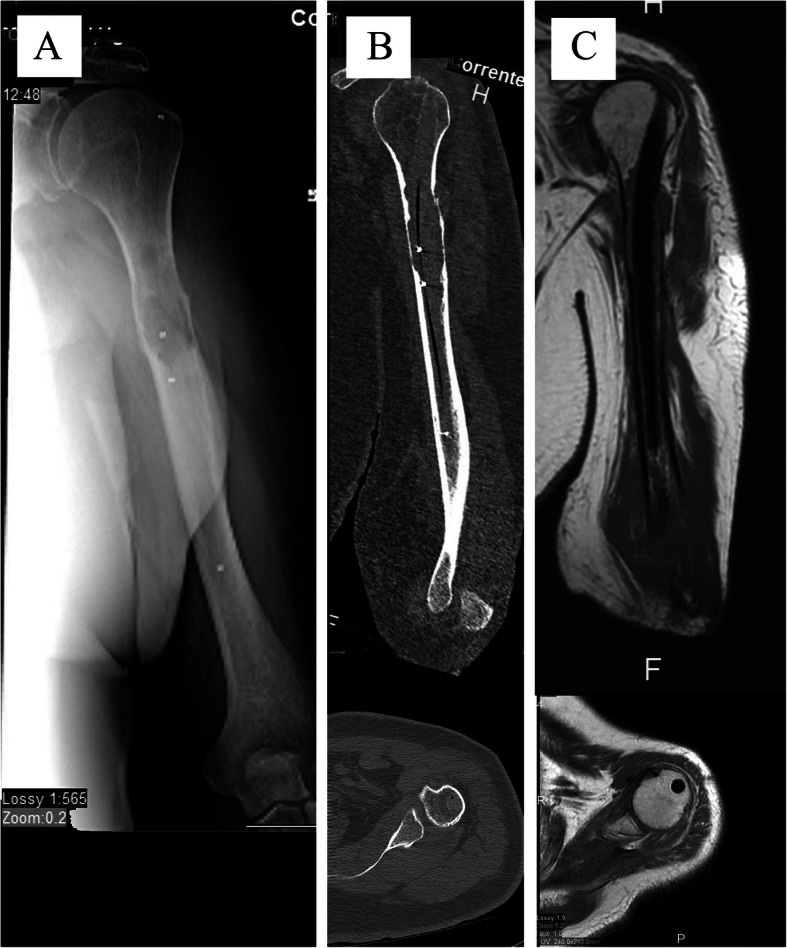
**a**: X-ray showing the IS-PBSS inside the humerus (the first nails only had a few markers and no information was available regarding the correct expansion; the latest models present a spiral marker which allows to view the correct expansion; **b**: the same patient’s CT-scan; the complete absence of artifacts enables easy detection of possible local recurrence and a better radiological irradiation; **c**: the corresponding MRI: note the absence of artifacts in this case also

We considered the reduction of radiolucency at X-ray for impending fracture and bone bridge formation between fragments at X-ray for already occurred fractures as signs of consolidation. A humeral CT-scan was performed for radiotherapy centering after about three weeks from surgery; in case a total body CT-scan was requested for oncological purposes to value systemic disease, the humerus was also added to the study and the imaging used to confirm consolidation (Fig. 2b); an MRI (Fig. 2c) was requested in case of suspected local progression. Supplementary controls were done on-demand of the oncologist.

The VAS scale was used to value pain before surgery and at one month of follow-up; the Musculoskeletal Tumor Society (MSTS) score was used to value the functional status preoperatively and at three months from the index surgery [15].

Iatrogenic fracture, intraoperative bleeding higher than 500 ml, nail rupture or polymerization problems, and neurological damage were considered intraoperative complications; loss of reduction, infections, hematomas, and wound dehiscence were considered postoperative complications.

Postoperative complications were distinguished in early or late if they occurred before or after 30 days from surgery, respectively [16].

An expert radiotherapist analyzed postoperative CT-scans to verify the presence/absence of artifacts related to the IS-PBSS and eventual problems for radiotherapy.

Statistical analysis: a descriptive statistical analysis was used to analyze data. The paired t-test was applied to value if the differences between preoperative and postoperative MSTS-score and VAS scale were statistically significant. A p < 0.05 was considered statistically significant. All analyses were performed using Microsoft Excel (Survival and local progression-free time were reported using Kaplan-Meier curves).

The authors declare the absence of any conflict of interest; no funding was received for the publication of the present paper.

Institutional Review Board approval was obtained for this study.

## Results

A series of 12 patients (for a total of 13 procedures), 7 males and 5 females, with an average age of 66 years old (min 51yo – max 77yo, SD 8.51) was included in our analysis; multiple myeloma was the most frequent histology, followed by lung cancer; all patients were addressed to orthopedic observation from medical oncology or hematology units where medical therapies were on-going; the epidemiological and clinical summary data are reported in Table 1.

**Table 1:** Global characteristics

Population	12 patients (seven males and five females)
**Average age**	66 years old (min 51yo – max 77yo, SD 8.51)
**Procedures**	• 12 unilateral procedures • One bilateral procedure
**Diagnosis**	• Multiple myeloma: 5 cases (one treated bilaterally); • Lung cancer metastasis: 3 cases; • Kidney cancer metastasis: 1 case; • Breast cancer metastasis: 2 cases; • Gastric cancer metastasis: 1 case *no solitary metastases were treated*
**Sites**	• Proximal humerus: 7 cases whereof 2 extended to the middle third • Middle third: 4 cases whereof one bilateral and 1 extended to the distal third; • Distal third: 1 case

### Indications for surgeries and fracture characteristics

 Four patients presented with impending humeral fractures with a Mirel’s score of 9 in three cases and 10 in the last case [4]. Seven cases presented with a fracture and the last one, affected by multiple myeloma, with a bilateral diaphyseal fracture and pseudarthrosis (Table 2). Five out of seven patients also presented with dislocation. The average preoperative VAS value was 7.

**Table 2:** Demographic and clinical characteristics

Pt	Age range	Side	Mirels score	Site	Histology	Defect length (mm)	F/I	Displaced	Standard nail possible?	Locking screw	Surgery length	Early compl.	Late compl.	Follow-up (months)	Status
**1**	76–80	R	n.v.	p.t.	Multiple Myeloma	96	f	y	y	prox	120’	n	n	32	a
**2**	71–75	R	9	p.m.t.	Lung cancer	116	i	.	n	n	59’	n	n	13	d
**3**	66–70	L	n.v.	m.t.	Clear-cell Kidney carcinoma	44	f	y	y	prox - distal	101’	n	n	16	d
**4**	51–55	Bilat	n.v.	m.t. (bilat)	Multiple Myeloma	L: 85; R: 103	f	y	n	n	210’	n	*	25	a
**5**	76–80	R	10	p.t.	Breast cancer	75	i	.	y	prox	75’	n	**	18	d
**6**	71–75	R	n.v.	p.t.	Lung cancer	59	f	n	y	prox	110’	n	***	17	a
**7**	61–65	R	n.v.	m.d.t.	Multiple Myeloma	37	f	n	y	distal	130’	n	n	60	a
**8**	61–65	L	9	p.m.t.	Multiple Myeloma	69	i	.	y	n	85’	n	n	24	d
**9**	71 − 65	R	n.v.	m.d.t.	Lung cancer	193	f	y	y	n	95’	n	****	16	d
**10**	66–80	R	n.v.	p.m.t.	Dediff. gastric cancer	77	f	n	y	n	70’	n	n	2	d
**11**	51–55	L	9	p.t.	Breast cancer	45	i	.	y	n	100’	no	n	27	a
**12**	51–55	R	n.v.	p.t.	Multiple Myeloma	42	f	y	y	n	80’	no	n	10	d

*M *male, *F *female, *n.v. *not valuable, *p.t. *proximal third, *p.m.t. *proximal-middle third, *m.t. *middle third, *m.d.t. *middle-distal third, *f *fracture, *I *impending fracture, *y *yes, *n *no, *a *alive with systemic disease, *d *dead for systemic disease progression; *: Left IS rupture at 12 months; **: local progression at 11 months; ***: traumatic nail procidentia at 11 months; ****: IS rupture at 4 months

The lesions presented an average length of 80.1 mm (median 75 mm, min 37 mm, max 193 mm). In seven cases, it was in the proximal humerus, whereof in three cases extended to the middle third; in six cases the lesion was in the middle third, whereof in two extended to the distal part.

The average preoperative MSTS score was 29.2% (11.7% in fractured patients).

### Surgical procedures

 A 2–3 cm incision was sufficient to insert the IS-PBSS in the humeral canal; a screw was inserted proximally to the disease in 3 procedures and distally in 1; in 1 out 13 procedures, two screws, proximal and distal, were inserted; no screws were needed in the remaining cases.

Reduction was obtained in all cases where a fracture was present except for one where a minimal 20-degree angulation remained.

In the case where bilateral inveterate pseudarthrosis was present, a residual angulation of about 15° was bilaterally present but considered acceptable without opening the fracture sites.

Surgeries lasted an average time of 95’ per site (min 59’, max 130’, median 100’) including the polymerization time (it varied between 600’’ and 1000’’ based on the nail size).

### Complications

All surgeries were carried out without intraoperative complications. The bleeding was not significant and no patients needed intra- or post-operative blood transfusions. No early postoperative complications were noted.

### Late complications

**Fig. 3:**
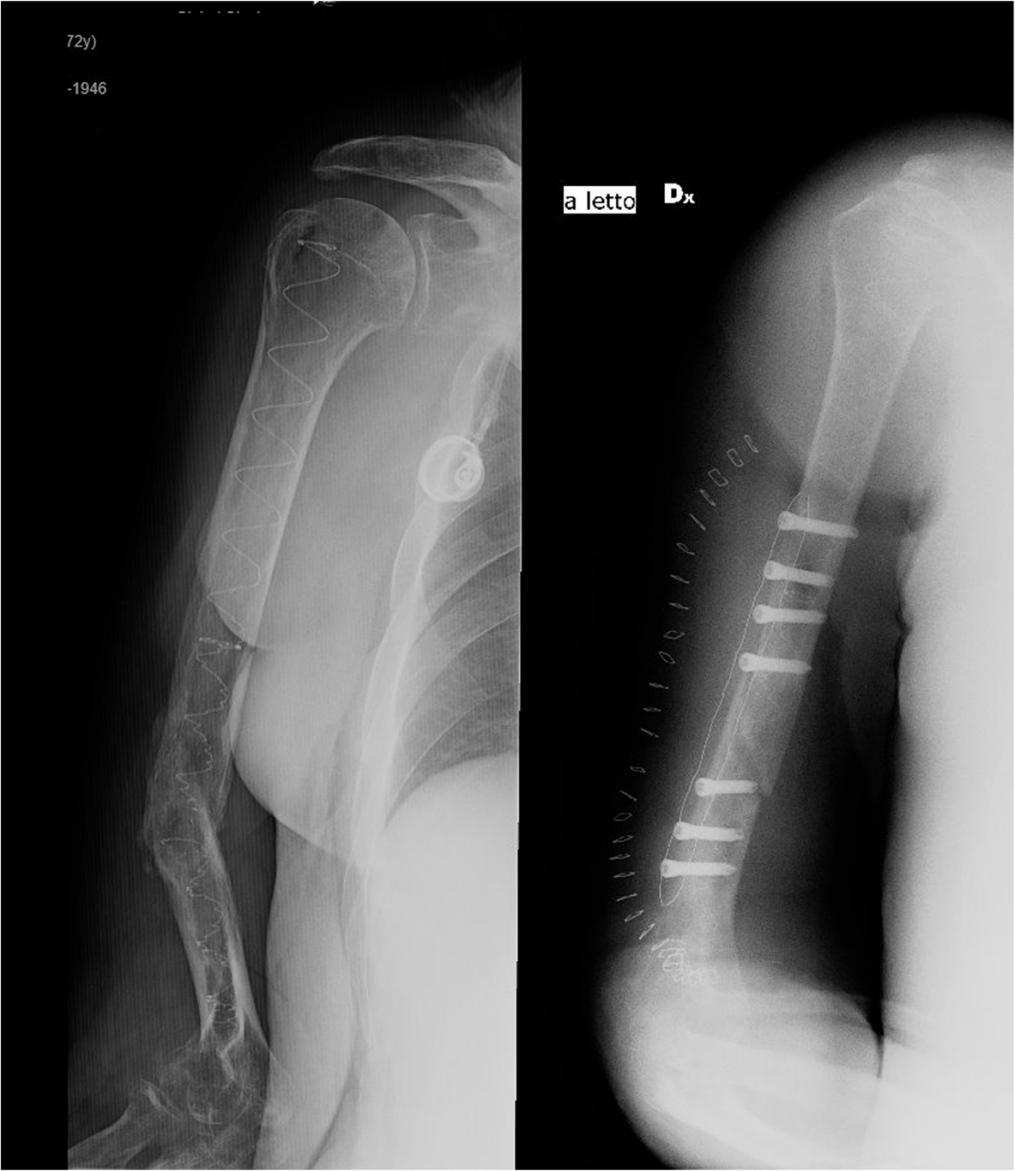
X-ray showing the rupture of the IS-PBSS on the left and the postoperative control after open reduction and plating; considering the poor bone quality, the IS-PBSS was maintained inside the canal as an augmentation to increase the screws’ effectiveness

 Two cases of nail ruptures were reported; the first one, in which a partial angulation was still present after surgery, underwent revision and plating for nail rupture at 4 months from surgery (Fig. 3) and the second one, affected by bilateral pseudarthrosis, ruptured the left IS-PBSS system after 12 months from index surgery, and was also resolved with plating.

One patient had proximal nail migration secondary to a trauma, 12 months post-operatively; he was treated with further surgery which consisted in cutting the protruding part of the nail since the stability provided by its remaining portion was deemed sufficient.

### Resumption of treatments

 The wounds healed in all cases; the patients were able to perform chemotherapy after three weeks. Radiotherapy was similarly performed; a wound dehiscence after irradiation was reported.

### Artifacts

No artifacts caused by the IS-PBSS were present at post-operative CT scans performed for identification of target areas for adjuvant RT. Minimal loss of information was reported around the screws in the cases where they were used to obtain greater stability.

### Functional recovery

At one month from surgery, all patients except for one had a painless active range of motion which reached 90°. The average postoperative VAS value was 2.6; the difference was statistically significant (*p* < 0.001). At three months of follow-up, function recovery was complete; the patients referred an occasional use of painkillers even though the assumption was often related to problems due to metastases in other sites. The average postoperative MSTS score was 87.8%; the difference was statistically significant (*p* < 0.001). Pain relief was probably also due to the radiotherapies and chemotherapies performed. The last patient still had pain in the fracture site, which worsened after four months. The IS-PBSS broke after another two months so he underwent new surgery and plating.

Consolidation was verified in seven out of 11 patients at 6 months of follow up (one patient died two months from surgery); in the bilaterally treated patient, consolidation was obtained on the right side at one year, while plating was necessary on the left side for IS-PBSS rupture; no real consolidation was radiologically evident in the other cases but the patients did not complain of any problems.

At six months of follow up, 11 patients (one bilaterally treated) were alive; one case had already been treated for IS-PBSS rupture.

### Survival

The survival rate was 91.7% at three and six months of follow-up, 83.3 and 41.7% at 12 and 24 months of follow-up, respectively; to date, at 66 months and 20 months from when the first and the last patient were enrolled, three patients are still alive: two affected by multiple myeloma at 32 and 60 months from index surgery and one affected by breast cancer at 27 months from index surgery.

All remaining cases have died from systemic disease progression without local disease progression, except for a woman, affected by breast cancer, who had disease nodules on her shoulder near the skin incision at 11 months from surgery, resolved with a surgical excision.

**Fig. 4:**
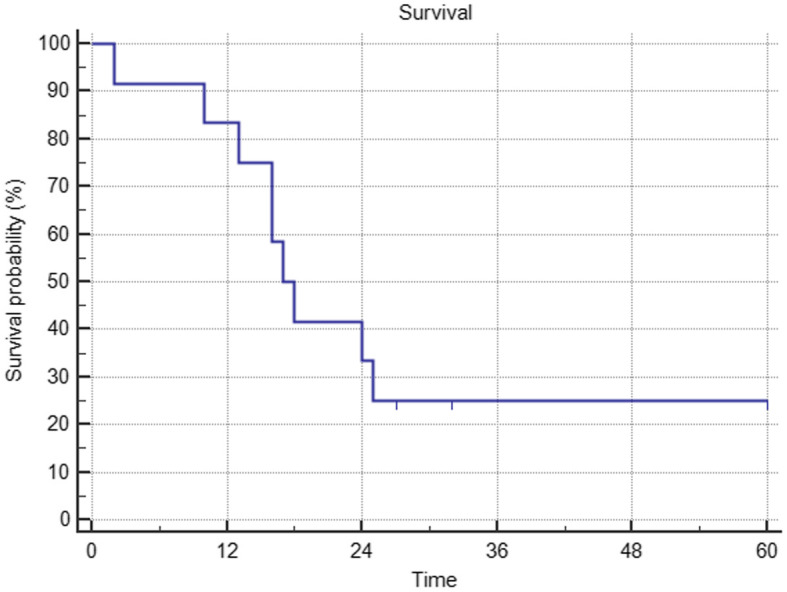
the Kaplan-Meier curve relative to the global survival of the present series

The Kaplan-Meier survival curve in shown in Fig. 4.

## Discussion

During the last decades, the improvement of medical therapies and the more efficient imaging techniques which allow a precocious diagnosis have produced a general increase of cancer survival, also in metastatic patients [17].

Previously, conservative treatment was preponderant for pathological osteolyses whereas surgery was reserved for pathological fractures; today, surgery is gaining more importance, also in the upper limbs, to assure a better quality of life [17].

Indeed, a fracture is a very negative event in the patient’s natural history because it often causes the interruption of medical therapies, indirectly influencing survival as well.

For this reason, research to find the best system to stabilize impending fracture and fracture continues.

The ideal surgical treatment in a metastatic patient should assure immediate pain control, functional recovery and a fast resumption of medical therapies and radiotherapies, interfering with these as less as possible. Therefore, the preference for minimally invasive techniques is evident. Several authors have published their experiences, highlighting the superiority of IM stabilization over open plating [18] so it is today considered the reference treatment for fractures or impending fractures located in the diaphysis in patients with an estimated survival inferior to 24 months affected by radiosensitive metastases from solid tumors. In case of localizations of hematological malignancies, IM stabilization is considering the reference treatment independently from the estimated survival.

IM stabilization is mainly performed with standard titanium locked nails, but titanium can cause a significant level of artifacts in advanced imaging studies (i.e. Computed Tomography, Magnetic Resonance Imaging, etc.) [19].

Titanium locked nails assure good biomechanical stabilization for diaphyseal fractures and impending fractures, but they are insufficient when the osteolysis becomes proximal or distal because of possible problems in inserting stable locking screws.

So, when the metastasis is too proximal, resection and prosthetic reconstruction is necessary, with the possible complications related to a bigger surgery.

The aim of IM stabilization is simply to provide biomechanical stability. An adjuvant treatment with radiotherapy is therefore needed to effectively halt local disease progression. Minimally invasive radiological techniques can also be useful to increase local control [20, 21]. Wide resection can sometimes be considered more appropriate in radioresistant tumors to decrease the risk of local progression.

Unfortunately, titanium nails also introduce artifacts at CT and MRI scans, with consequent difficulties during follow-up and especially for postoperative radiotherapy [19].

Recently, carbon fiber nails have become commercially available. The stability they assure is comparable to titanium nails but without giving artifacts, so they should assure an easier and safer adjuvant radiotherapy. However, they have the same limits regarding proximal osteolyses and fractures [19].

On the contrary, IS-PBSS conforms to the unique and specific anatomy of the medullar canal, stabilizing the bone and also giving rotational stability by adhering to the internal cortical surface; it does not need locking screws and can be used for proximal and distal lesions as well. Moreover, in case it is judged insufficient, IS-PBSS is easily drilled and stabilized by one or more interlocking screws, as done in our series. However, this is not so common: in the present series, screwing occurred more often in first cases we performed, when we were not so confident in the technique and in the ability of IS-PBSS to stabilize the segment. In our opinion, screwing should be avoided when possible because a possible reduction in IS-PBSS strength has to be considered.

### Indications

- Impending fracture: the site, the geometry of the osteolysis and the bone quality also have to be valued; as already said, when the osteolyses is extremely proximal, IM traditional nailing can be not possible because the proximal locking screws can be not able to stabilize the segment; for the same reason, sometimes, the presence of other lesions in the same segment is a contraindication for nailing, as low bone quality; in these cases, IS-PBSS may assure a higher stabilization than standard. Indeed, IS-PBSS particularly shows its utility in lesions located in the epiphysis, where it can be considered a valid alternative to prostheses for metaphyseal lesions in not long-survivors.
- Already occurred fracture: IS-PBSS can be also used but it could underwent excessive mechanical stress, moreover if a consistent loss of bone and related instability is present; indeed, it can be difficult to maintain reduction during the entire lasting of polymerization.

In case of fracture it is suggested to set a table under the forearm useful to maintain the arm during polymerization; we maintain the forearm fully supinated on the support to decrease the risk of rotational error.

### Advantages

 Based on present experience, IS-PBSS could be considered even more minimally invasive than traditional IM nails because a small extracapsular incision is sufficient for introduction in most cases, even when the entry point is not entirely in line with the anatomical axis of the humerus due to the malleable, non-solid state in which the monomer is inserted into the IM canal.

This allows us to spare the rotator cuff with a direct advantage and fast recovery. Indeed, in our series all patients were able to resume their preoperative therapies after less than 3 weeks from surgery, a quite complete function at one month with a statistically significant increase of the MSTS-score and a reduction of the postoperative VAS. Actually, also no postoperative wound dehiscences were reported, even if one case it occurred after radiotherapy.

Because of its radiolucency, IS-PBSS eliminates artifacts in CT- and MRI-scans. These characteristics allow an easier follow-up, a better visualization of the fracture’s healing process, an early detection of tumor recurrence or local progression during follow-up, even at the bone-muscle interface which is usually hidden by the titanium artifacts, but mostly a better target volume contouring, and an easier and safer radiotherapy by optimizing the dose volume planning (Fig. 2A and B) [1].

In the present series, no early complications were reported; blood loss can be considered very low, probably because of the possibility to insert the IS-PBSS without reaming. In a recent paper, Yu et al. demonstrated how bleeding is directly related to the ratio between nail diameter and medullary canal diameter [22]. In this sense, the IlluminOss system can be considered the optimum because when the nail is inflated, it adheres to the intramedullary cortical surface, supplying direct compression and hemostasis.

Indeed, no patient needed blood transfusion during surgeries nor during the postoperative period.

### Disadvantages and complications

 Two IS-PBSS ruptures verified at 4 and 12 months of follow-up; although they could be due to erroneous indications or incorrect surgical technique, the real strength of the system has to be clinically proven in a more numerous series.

Nevertheless, Hoellwarth et al. recently published a paper in which they reported IS-PBSS rupture in 4 out of 19 cases, confirming our data; they also verified no statistical difference in reoperation rate and hardware durability among IS-PBSS, standard titanium nail and cemented plate in a cohort of 105 pathologic humeral fracture [23].

A possible related hot topic can be considered its removal in case of synthesis failure even after years, due to nonunion or delayed union, local disease progression with subsequent breakage of the system or its mobilization, or infection.

Even if MC Sweenay et al. published a study performed on rabbits in which they demonstrated the perfect compatibility of the IS-PBSS inside active bone without any significant tissue reaction and its integration, a possible limitation in the removal procedure is represented by the unique shape the nail can assume inside the patient’s bone; this can make the procedure extremely aggressive from a surgical point of view. [24]

The infection rate seems very low in literature; it could be due to the minimal invasiveness which reduces the risk of contamination although, recently, a direct antimicrobial effect of the blue-light applied to induce polymerization was hypothesized [25].

Also, other possible complications have to be considered, as the damaging of the IS-PBSS shell during insertion in the medullary canal and the consequent spreading of the monomer into the tissues or possible radial nerve lesions due to the exothermic reaction even if neither have been noticed in the present series nor in literature .

This paper obviously presents several limitations, such as the retrospective design, the very limited number of cases, the heterogeneous population with both impending fractures and fractures; nevertheless, based on our experience, IS-PBSS is a valid alternative to standard nails, especially for frail patients, because of its low invasiveness and easy technique of insertion; it could be considered the first choice in compromised patients when the lesion is located in the epiphysis and standard nails cannot be locked.

## Conclusions

Impending and pathological fractures of the humerus pose a challenge to the orthopedic surgeon, due to the nature of the fracture per se, and to the patient’s poor conditions. When surgical treatment is indicated, it must provide an adequate stabilization through a minimally-invasive technique. Moreover, the use of a technology which is compatible with further oncological diagnostic procedures and baseline therapies (i.e. radiotherapy) must be taken into account. In these terms, the IS-PBSS can be considered a valid alternative in osteolyses from solid tumors or localization of myeloma in the humerus; although it can be used in already occurred fracture, our suggestion is to use it especially for impending fractures, mostly when the cortical erosion is consistent and it is not possible to insert locking screws in a standard nail. It represents a good alternative to resection and prosthetic reconstruction in case of epiphyseal lesions, moreover in frail patients.

The easy surgical technique, the minimal invasiveness and related fast recovery and pain control, and the system’s radiolucency can be considered the main advantages. Indications have to be valued considering a limited strength and a possible loss of correction during polymerization in case of unstable fractures. Moreover, more clinical data are necessary to value the actual strength of the system.

Further clinical studies must be performed to verify outcomes in terms of rate of fracture healing, complications, nail failure, patient and implant survival and clinical benefits in radiotherapy.

## Data Availability

All patients’ data are available in the corresponding hospitals.

## Abbreviations

IM: intramedullary
IS-PBSS: IlluminOss® photodynamic Bone Stabilization System
